# Sub-100 fs Formation of Dark Excitons in Monolayer WS_2_

**DOI:** 10.1021/acs.nanolett.4c03807

**Published:** 2024-11-08

**Authors:** Pavel V. Kolesnichenko, Lukas Wittenbecher, Qianhui Zhang, Run Yan Teh, Chandni Babu, Michael S. Fuhrer, Anders Mikkelsen, Donatas Zigmantas

**Affiliations:** † Institute of Physical Chemistry, Heidelberg University, 69120 Heidelberg, Germany; ‡ Institute for Molecular Systems, Engineering and Advanced Materials, Heidelberg University, 69120 Heidelberg, Germany; ¶ Division of Chemical Physics, Lund University, P.O. Box 124, 221 00 Lund, Sweden; § NanoLund, P.O. Box 124, 221 00 Lund, Sweden; ∥ Department of Physics, Lund University, Box 118, 221 00 Lund, Sweden; ⊥ Department of Civil Engineering, Monash University, Melbourne, Victoria 3800, Australia; # Centre for Quantum Science and Technology Theory, Swinburne University of Technology, Melbourne, Victoria 3122, Australia; @ School of Physics and Astronomy, Monash University, Melbourne, Victoria 3800, Australia; △ ARC Centre of Excellence in Future Low-Energy Electronics Technologies, Monash University, Melbourne, Victoria 3800 Australia

**Keywords:** monolayers, excitons, 2D materials, femtosecond, photoemission, microscopy

## Abstract

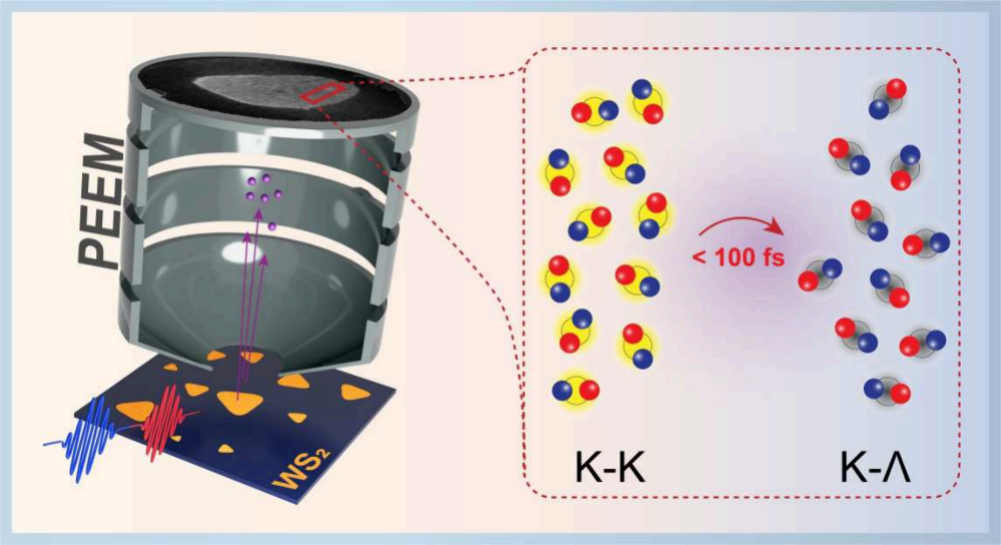

Two-dimensional semiconducting transition metal dichalcogenides are promising materials for optoelectronic applications due to their strongly bound excitons. While bright excitons have been thoroughly scrutinized, dark excitons have been much less investigated, as they are not directly observable with far-field spectroscopy. However, with their nonzero momenta, dark excitons are significant for applications requiring long-range transport or coupling to external fields. We access such dark excitons in WS_2_ monolayer using transient photoemission electron microscopy with subdiffraction limited spatial resolution (75 nm) and exceptionally high temporal resolution (13 fs). Image time series of the monolayer are recorded at several different fluences. We directly observe the ultrafast formation of dark K-Λ excitons occurring within 14–50 fs and follow their subsequent picosecond decay. We distinguish exciton dynamics between the monolayer’s interior and edges and conclude that the picosecond-scale evolution of dark excitations is defect-mediated while intervalley scattering is not affected by the defects.

Two-dimensional (2D) semiconductors such as monolayers of transition metal dichalcogenide (TMdC) are promising for applications in optics and optoelectronics because they exhibit rich exciton physics.^1,2^ The large exciton binding energies of ∼0.5 eV in these materials make 2D excitons stable at room temperature, allowing for high exciton densities.^3^ This has enabled observations of various exciton formations including neutral^4^ and charged^5^ excitons, biexcitons,^6^ and higher-order exciton complexes.^7,8^ This, in turn, renders TMdC monolayers an ideal platform for investigating various many-body interactions and related emerging phenomena.^9−13^

Excitons with zero momentum form within TMdC monolayers in K valleys of their Brillouin zone, facilitated by enhanced electron–hole Coulomb interactions.^14^ These excitons have been thoroughly investigated in the literature^15^ because they form within the light cone in energy-momentum space enabling their straightforward spectroscopic interrogation. Subsequent scattering of carriers to adjacent valleys leads to the formation of indirect excitons with nontrivial momenta falling outside the light cone,^16^ rendering them optically dark. Due to their net nonzero momentum, studying dark excitons is of importance because they can be better alternatives to bright excitons for long-range transport^17−23^ or coupling to external fields such as those induced by plasmons.^24,25^ Accessing nontrivial-momentum dark excitations, however, is an experimental challenge: optical spectroscopy cannot directly probe them, and momentum-resolved photoemission spectroscopy, although capable of directly detecting intervalley carriers, is experimentally highly challenging.^26^ In addition, it does not provide subdiffraction-limited spatial resolution,^27^ which is often highly desirable. Nevertheless, photoemission-based spectroscopies remain essential for directly accessing dark excitons, which cannot be probed directly by light because of the momentum-forbidden transition.

Many studies have reported on the life-cycle of 2D excitons in various experimental settings covering their formation,^28−30^ ultrafast cooling,^31^ intervalley scattering,^16,32−34^ and ultimate fate (e.g., exciton–exciton annihilation,^17,20,35^ electron–hole recombination,^20^ and exciton dissociation^36^). Exciton formation, cooling, and intervalley scattering are among the initial processes occurring on femtosecond time scales^37^ with the fastest reported being on a sub-100 fs time scale.^30,32,33^ Therefore, in studying dark excitons, an additional challenge of resolving early stage dynamics must be overcome–advanced ultrafast spectroscopy techniques with very high temporal resolution are required. However, pushing the resolution of femtosecond photoemission-based apparati to the level needed for confident identification of sub-100-fs ultrafast processes poses a formidable challenge.

Previously, femtosecond carrier kinetics in TMdC materials have been investigated using optical and photoemission-based spectroscopies with time resolutions in the range of 20–200 fs with the most common value being a few tens of femtoseconds.^16,28−33,38−45^ In particular, sub-100-fs carrier dynamics have previously been identified in MoS_2_,^30^ WSe_2_,^29,33^ and WS_2_^16^ monolayers. In the latter case, an intervalley transfer time of 16 ± 5 fs was reported from temporal offsets of momentum-resolved signals via two-photon photoemission with >50 fs temporal resolution. In this study, we pushed the time resolution below 20 fs enabling straightforward observation in the temporal domain of sub-100 fs formation of momentum-forbidden dark excitons in monolayer WS_2_, measured in a simpler setting via one-photon photoemission from the exciton states.

More specifically, we combine photoemission electron microscopy (PEEM) with high spatial resolution^46−48^ (75 *nm*) and femtosecond pump–probe spectroscopy with exceptionally high temporal resolution (13 fs). By pumping at the main exciton resonance (2 eV), we study subsequent carrier dynamics in WS_2_ monolayer using the transient PEEM (TR-PEEM) apparatus. By design of the experiment, we detect ultrafast intervalley scattering from K valleys (i.e., the formation of dark excitons) occurring with a time constant in the range of 14–50 fs, detected via photoemission stimulated by broadband probe pulses in the deep-ultraviolet (DUV, 4.7 eV) region. High temporal resolution was ultimately achieved by generating very short DUV pulses via achromatic phase matching in a nonlinear crystal, a method described previously^49,50^ but never used before as part of transient photoemission electron microscopes. We finally take advantage of the imaging capabilities of the setup to additionally distinguish spatially heterogeneous signals from the monolayer interior and edges, pointing at long-term defect-mediated processes.

The experiment is shown in Figure 1a (see Supporting Information, Section S1, for more details). Prior to measuring carrier dynamics in the WS_2_ monolayer, we first verified that the intensity of the pump beam was low enough to avoid high-order pump-induced photoemission from the flake (see Supporting Information, Section S2). For the pump fluences used in our experiments, excitation density was estimated to be of the order of 10^11^*cm*^–2^ which is 3 orders of magnitude lower than the Mott density.^3^ This additionally ensures that the dominant pump-generated carriers are indeed bound electron–hole pairs (excitons).^51^ The energy of the probe pulses ultimately determined the photoemission horizon^31^ in the energy-momentum space (Figure 1b)
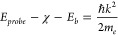
1beyond which no photoemission is possible. In eq 1, *E*_*probe*_ is the probe pulse energy, χ is the effective electron affinity of monolayer WS_2_, *E*_*b*_ is the exciton binding energy, *m*_*e*_ is the mass of electron, and *k* is the in-plane momentum. As seen in Figure 1b, the detectable photoemission signal can only originate from the region (shaded blue) energetically higher than the photoemission horizon covering momenta in the vicinity of the Γ point of the Brillouin zone, so that the valleys at the ±Λ points, which are energetically close to the conduction band minima, can contribute photoemitted electrons via K-to-Λ intervalley scattering. Such scattering is expected to be energetically favorable in WS_2_ monolayers^16,34,52,53^ and enhanced in *n*-doped monolayers^54^ leading to efficient generation of dark excitons. Additionally, the inherent presence of S-vacancies on the flake results in midgap states 0.3–0.5 eV below the conduction band^55,56^ that can also fall into the probe region.

**Figure 1:**
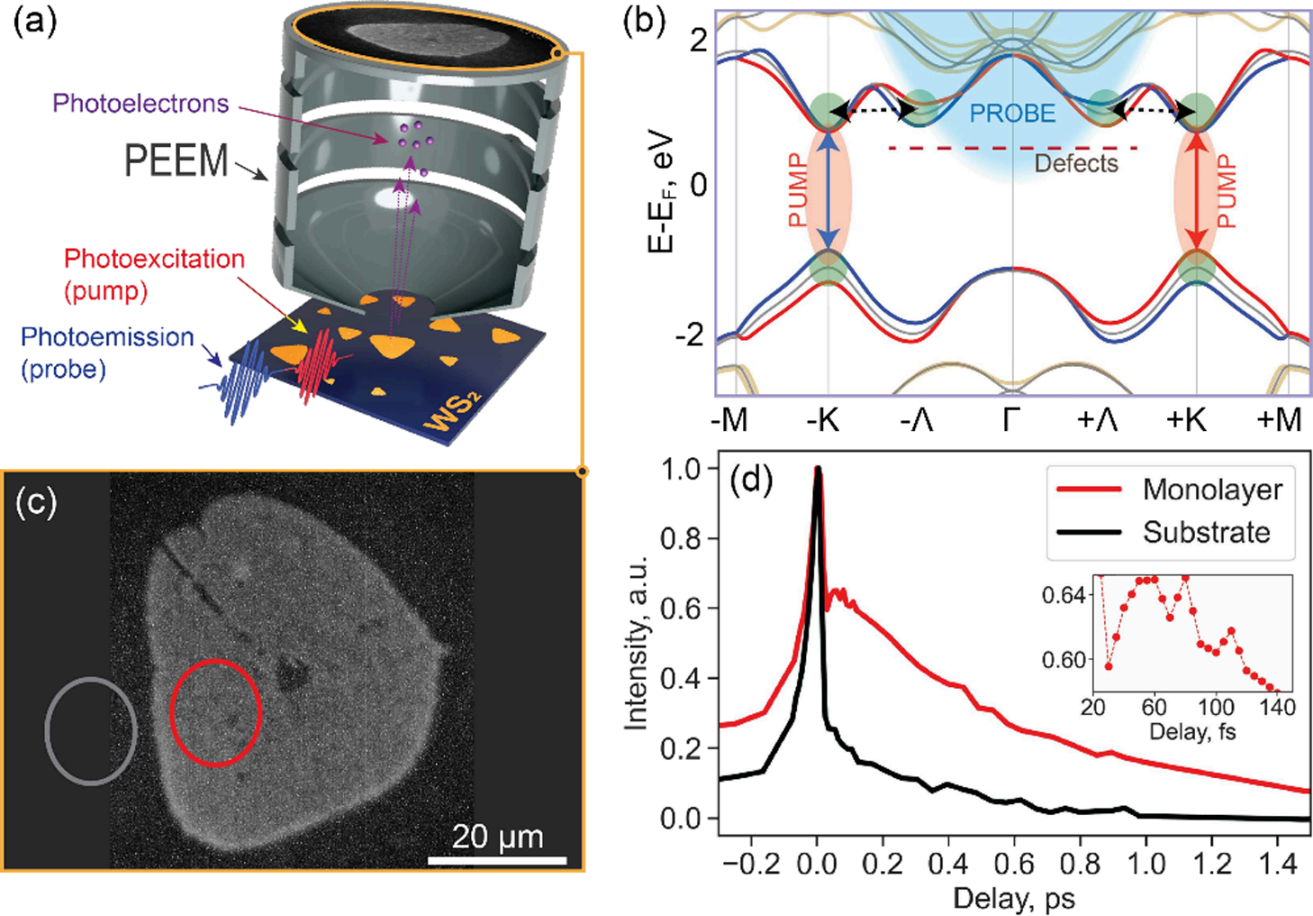
(a) A TR-PEEM experiment. Pump pulse (red) excites carriers in monolayer WS_2_ (orange); probe pulse (blue) photoemits electrons (purple) from the monolayer; these electrons form an image (encircled with orange line) for each pump–probe delay. (b) Band structure of WS_2_ monolayer calculated using DFT (Quantum Espresso^57^). Exciton transitions are indicated by blue and red arrows. Intervalley scattering is represented by dashed arrows. The area above the photoemission horizon is schematically shown shaded in blue with a Gaussian edge reflecting the final bandwidth of DUV pulses. (c) An image of a WS_2_ monolayer flake obtained using a Hg (mercury) discharge lamp. The length of the scale-bar is 20 μm. The gray and red circles indicate areas, across which signals in (d) were integrated for each pump–probe delay. (d) Normalized pump–probe traces spatially integrated across the monolayer-interior (red) and substrate (black) regions shown in (c). The inset shows sub-100 fs rise and decay of the photoemission signal. Pump and probe fluences were 140 nJ/cm^2^ and 70 nJ/cm^2^ per pulse, respectively, and integration time was 10 s.

A photoemission image of a typical WS_2_ monolayer flake is shown in Figure 1c. Similar flakes have been characterized previously^58−60^ revealing the energy of the main exciton resonance to be ∼2 eV and that of charged excitons (trions) to be a few tens of meV (a charging energy) below. In TR-PEEM experiments reported here, the energy of the pump pulses was tuned to overlap the main exciton resonance thus promoting adiabatic exciton formation.^16,30^ The broad spectrum of the pump pulses also overlapped with the energy of the trions. The resulting coherent exciton polarization is expected to lose its coherence within 100 fs^16,30^ giving rise to incoherent excitons and trions. Pump-induced dynamics were then monitored via probe-induced photoemission. By the construction of our experiment, TR-PEEM is sensitive to dark intervalley carriers, which are not directly accessible by conventional optical spectroscopies (although, their dynamics may be deduced indirectly via modeling^61^). Measured typical pump–probe traces from the substrate and the interior of the monolayer WS_2_ are shown in Figure 1d. Similar traces were also observed from other flakes on the substrate (not shown here). In contrast to the substrate, the monolayer WS_2_ features a prominent well-resolved rise of the photoemission signal on a sub-100-fs time scale.

Before conducting a more detailed analysis of the observed carrier dynamics, it is necessary to assess whether the substrate contributed to the photoemission background in the measured pump–probe signals across the monolayer flake. The work function of Si and SiO_2_ have been previously reported to be 4.8 eV and 4.4 eV, respectively.^62^ Given the pump (2.0 eV) and probe (4.7 eV) energies used in this work, it is likely for substrate-electrons to be photoemitted from at least the SiO_2_ layer with excess energy *E*_*e*_ (electron kinetic energy) in the range of ∼0.3–2.3 eV. The de Broglie wavelength λ_*e*_ of such electrons is estimated as  (*m*_*e*_ is electron’s mass, and *h* is Planck’s constant) yielding the values in the range of 0.81–1.22 nm, which are larger than the thickness ∼0.6 nm of WS_2_ monolayers.^63^ The inelastic mean free path of such electrons within the monolayer is expected to be greater than 80 nm in accordance with the universal curve for inorganic compounds,^64^ exceeding the monolayer’s thickness by 2 orders of magnitude. Therefore, a substantial photoemission background from the substrate is indeed expected to contribute to the detected transient photoemission signals obtained from the monolayer region. We thus use photoemission directly from the substrate as an estimate for such background and subtract it from the overall photoemission signal in a manner that naturally suppresses coherent contributions in pump–probe traces (see Supporting Information, Section S3, for more details). This procedure also ensures that substrate effects such as surface space charge region, surface dipoles, surface carrier recombination, and surface state distribution,^65^ which are not or only weakly affected by the monolayer, are also taken into account in the further analysis. The retrieved transient WS_2_-specific photoemission contrast is shown in Figure 2. As expected, these differential dynamics feature a delayed rise of the photoemission signal followed by its subsequent decay. To gain more insights into the underlying carrier dynamics, we applied a simple fitting model to the resultant traces (see Supporting Information, Section S4), which takes into account the finite rise of the detected signal^30,66^
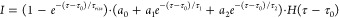
2where τ_*rise*_ is the time-constant describing the sub-100-fs photoemission build-up; τ_1,2_ describe subsequent dynamics beyond 100 fs; *a*_1,2_ are corresponding fitting amplitudes; *a*_0_ and τ_0_ are constant photoemission offset and time-zero calibration, respectively; *H* is the Heaviside step function.

**Figure 2:**
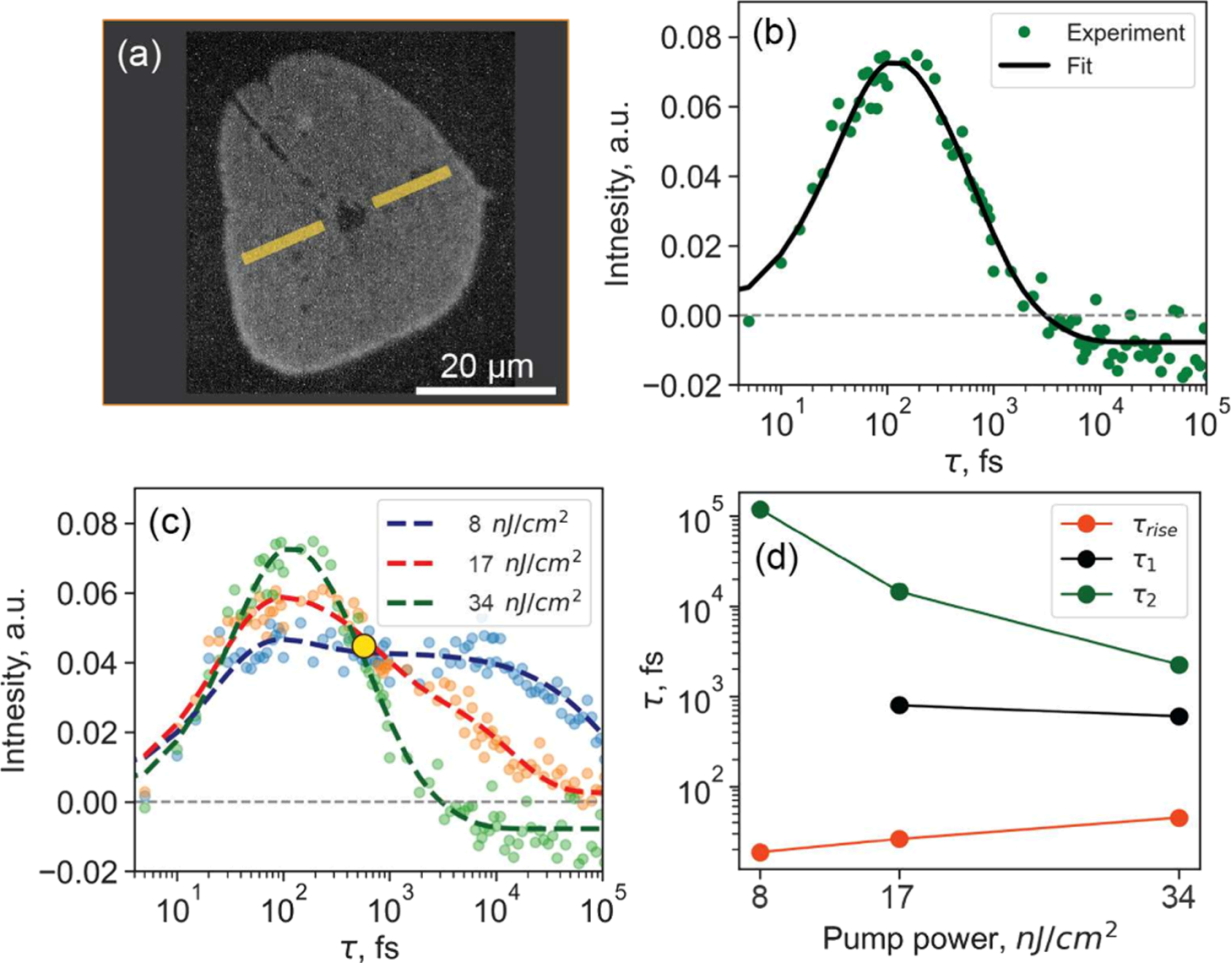
(a) Area (yellow), across which signal was integrated for analysis. (b) Fitting of the pump–probe trace obtained by integrating the pump–probe signal across the area (indicated in (a)) on WS_2_ flake. Pump fluence was 34 nJ/cm^2^. (c) Pump–probe traces (dots) and corresponding fits (dashes) from the same area as in (a) acquired for pump fluences of 8 (blue), 17 (red), and 34 (green) nJ/cm^2^. Isometric point is indicated by yellow circle. (d) Power-dependence of extracted time-constants τ_*rise*_ (red), τ_1_ (black), and τ_2_ (green). The first τ_1_ point is not shown since it does not correspond to the second rising signal (mentioned in the main text).

Figure 2b shows the result of fitting applied to a pump–probe trace obtained with 34 nJ/cm^2^ pump pulses, featuring an adequate fitting quality. We note a negative offset at longer delays indicating relative photoemission from the flake is less than that from the substrate, which we attribute to the effects of bandgap renormalization.^67−72^ Such renormalization could result in Λ-valleys lowering their energy below the photoemission horizon and/or less energetically favorable scattering to the probe region (if K-valleys become lower in energy than Λ-valleys). Regardless of these two possible mechanisms, the net effect from both of them is a reduction in the photoemission signal for higher pump fluences. To provide support for the hypothesis of the bandgap renormalization effects, we measured pump-induced photoemission for higher pump fluences resulting in an even weaker photoemission signal (see Supporting Information, Section S5).

The retrieved time-constants are τ_*rise*_ ∼ 45 fs, τ_1_ ∼ 0.6 ps, and τ_2_ ∼ 2.2 ps, which are similar to those of previously reported processes in 2D TMdC-based semiconductors, namely, (τ_*rise*_) exciton formation^30,33^ and K-to-Λ intervalley scattering,^16,33^ and (τ_1,2_) scattering away from Λ-valleys,^33,73,74^ exciton decay,^28,42^ exciton–exciton annihilation,^33^ and the formation of trions.^66^ Due to the very low excitation densities used in our experiments, we exclude exciton–exciton annihilation processes. Geminate excitons form outside the probe region in the energy-momentum space and therefore should not contribute to photoemission either. In addition, their formation should be independent of pump fluence, but scattering processes are, in contrast, pump-fluence dependent. In the latter case, we note that for such low excitation densities used in this work, exciton–exciton scattering is not a plausible intervalley transfer mechanism; rather, it is scattering mediated by phonons^52,75^ and/or plasmons^76^ (due to intrinsic *n*-doping of monolayers grown via chemical vapor deposition (CVD)^77^) that are likely at play in this case. To confirm the scattering processes taking place and gain further insight into the detected sub-100-fs photoemission rise and subsequent dynamics, we acquired pump-fluence dependences of pump–probe signals (Figure 2c,d).

Figure 2c shows pump–probe dynamics measured for three pump fluences of 8 nJ/cm^2^, 17 nJ/cm^2^, and 34 nJ/cm^2^. Notably, fluence-dependent changes of signal amplitudes occur in opposite directions for delays shorter and longer than ∼700 fs. Specifically, for delays <700 fs photoemission intensity increases with pump fluence, whereas for delays >700 fs photoemission intensity decreases. This behavior indicates a possible interplay of competing processes, which is also supported by the observation of a second rising signal (peaking at ∼7 ps) most prominent in the pump–probe dynamics obtained with the lowest pump fluence of 8 nJ/cm^2^ (see also Supporting Information, Section S4). This observation points to a possible sequential carrier-transfer phenomenon that becomes more dominant for low pump fluences. The delay of ∼700 fs, in this case, can be regarded as an isometric point^78^ in the temporal domain, i.e., the point at which the signal is independent of pump fluence (which is in agreement with nearly constant τ_1_ for higher pump fluences). We note that the chosen model (eq 2) fits well the two higher-fluence pump–probe traces, but does not explicitly take into account this secondary carrier transfer observed in the low-fluence case. Instead, by applying a fitting model that takes into account two rising and two decaying signals (see Supporting Information, Section S6), we can obtain a better fit to the pump–probe trace for the lowest fluence. In this case, signal rise times of τ_*rise*,1_ ∼ 23.7 fs and τ_*rise*,2_ ∼ 1.1 ps are retrieved, which are in agreement with the reported values of intervalley exciton scattering^16,33^ and trion formation,^66^ respectively. In Figure 2c, nevertheless, the fluence-dependent trends are extracted using the model described by eq 2 for consistency.

All three time constants, overall, are fluence-dependent (Figure 2d) indicating contributions from scattering-mediated processes, with τ_*rise*_ increasing, and τ_2_ decreasing with pump fluence. Initially, the time-constant τ_1_ increases and then mildly decreases for increasing pump fluence (see Supporting Information, Section S4), which also supports an interplay of competing processes mentioned above. A lower scattering rate, as indicated by larger τ_*rise*_, for larger pump fluences further fortifies the notion that there are additional effects of bandgap renormalization at play. The decreasing trend for τ_2_ also additionally supports the above-mentioned second signal rise, which in this case likely indirectly reflects scattering away from Λ-valleys or directly reflects decay to longer-lived defect states (such as those introduced by S-vacancies) and/or generation of trions. Further studies are required to disentangle all these effects. Given the discussion above, the time-constant τ_1_ reflects an interplay between exciton decay to lower states (such as traps and trions) within the probe region, and carrier scattering away from the probe region. Thus, for lower fluences, it is the exciton decay to longer-lived states that is prevalent resulting in lower rates and a prominent second rise of photoemission. For higher fluences, when bandgap renormalization takes place, energetically lowered K-valleys could result in a more efficient backscattering.

We finally note that the measured carrier dynamics are most likely defect-mediated. It has been observed previously that edges in CVD-grown WS_2_ monolayers contain larger amount of S-vacancies compared to the interior.^55,79,80^ Defect densities in the interior can be as large as ∼10^13^ cm^–2^, whereas those near the edges–of the order of ∼10^14^ cm^–2^,^55^ which are 2–3 orders of magnitude larger than the excitation densities used in this work (see also ref (79)). Therefore, we next take advantage of the spatial resolution afforded by the method, and directly compare pump–probe dynamics from the interior (with a lesser number of S-vacancies) and edges (with a larger density of S-vacancies) of the monolayer flake. Figure 3 shows pump–probe dynamics from the interior and edges for the three investigated pump fluences. In all cases, a slower decay dynamics from the edges is evident confirming contributions from trap-mediated exciton dynamics across the flake. Notably, the intervalley scattering rates are not as different between the interior and edges of the monolayer (see Supporting Information, Section S4) indicating that defects likely do not act as efficient scattering centers in this case. Nevertheless, compared to the interior, at the edges there is a clear trend of a lower sub-100 fs photoemission signal accompanied by larger photoemission on picosecond time scale (with photoemission intensity unchanged at subpicosecond delays) similar to what was observed in fluence-dependent measurements. This further supports that the prevailing decay to longer-lived states for lower pump-fluences discussed above is indeed in part the decay to defect states.

**Figure 3:**
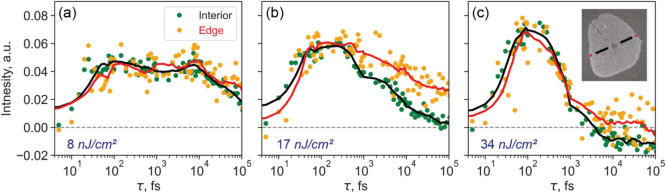
Comparison of pump–probe signals acquired from interior (green dots and black lines) and edges (orange dots and red lines) of the WS_2_ flake for three excitation powers of (a) 8, (b) 17, and (c) 34 nJ/cm^2^. Solid lines represent the result of smoothing to reveal fine details of the traces. Signal integration areas are indicated in the inset shown in (c).

Following the discussion above, it can be concluded that, within the range of set experimental conditions, dark excitons in monolayer WS_2_ form within ∼14–50 fs – in excellent agreement with intervalley scattering rates in TMD materials reported previously^16,45^ – followed by picosecond-scale dynamics mediated by defects. This ultrafast formation of dark excitons occurs as fast as the cooling of bright excitons^30^ suggesting that dark-exciton configuration is more energetically favorable in WS_2_ monolayers. We finally note that other contributions such as the bright-exciton formation, intervalley scattering of spin-forbidden dark excitons, and cooling of doping electrons are not expected to contribute significantly, and that the developed TR-PEEM approach is sensitive predominantly to dark excitons (see Supporting Information, Section S7).

In summary, we investigated intervalley carrier dynamics in monolayer WS_2_ via PEEM coupled to femtosecond pump–probe spectroscopy with a very-high temporal resolution of 13 fs. We identified initial K-to-Λ intervalley scattering (formation of dark excitons) occurring on a time scale of 14–50 fs, depending on the excitation and defect density. The intervalley scattering does not appear to differ significantly between the edges and the interior of WS_2_ monolayer. Subsequent dynamics suggested a decay of dark excitons to longer-lived states. A defect-mediated dynamics at the monolayer edges were unambiguously identified by taking advantage of the imaging capabilities of the apparatus with a subdiffraction-limited spatial resolution of 75 *nm*. The developed ultrafast spectro-microscopy approach can be used for direct identification of sub-100-fs processes in other TMdC monolayers as well as graphene^81^ and other topological semimetals.^82^ Furthermore, complementing the ultrafast TR-PEEM method with excitation frequency resolution^38^ and energy resolution of the photoemitted electrons as well as momentum resolution will provide a more comprehensive picture of the ultrafast processes taking place in these materials.

## Supporting Information Available

Methods, pump-induced excitation and photoemission, monolayer-specific dynamics, fittings of pump–probe traces, photoemission contrast drop for higher pump fluences, fitting with two rise-and-decay signals, and comment on other possible effects (PDF)
