# Supplementary material for: Sub-100 fs Formation of Dark Excitons in Monolayer WS2
Source: Nano Lett. 2024 Nov 8;24(46):14663–70. doi: 10.1021/acs.nanolett.4c03807 (PMC11583335; doi:10.1021/acs.nanolett.4c03807)
Supplement: Supplementary file 1 — nl4c03807_si_001.pdf [file nl4c03807_si_001.pdf]

Supplementary material to: "Sub-100-fs formation of dark excitons in monolayer WS_2"

Pavel V. Kolesnichenko,[*,†,‡,¶,§] Lukas Wittenbecher,[‖,¶,§] Qianhui Zhang,[⊥] Run Yan Teh,[#] Chandni Babu,[¶,§] Michael S. Fuhrer,[@,△] Anders Mikkelsen,[‖,§] and Donatas Zigmantas[*,¶,§]

†*Institute of Physical Chemistry, Heidelberg University, 69120, Heidelberg, Germany*

‡*Institute for Molecular Systems, Engineering and Advanced Materials, Heidelberg University, 69120, Heidelberg, Germany*

¶*Division of Chemical Physics, Lund University, P.O. Box 124, 221 00 Lund, Sweden*

§*NanoLund, P.O. Box 124, 221 00 Lund, Sweden*

‖*Department of Physics, Lund University, Box 118, 221 00 Lund, Sweden*

⊥*Department of Civil Engineering, Monash University, Melbourne, Victoria 3800, Australia*

#*Centre for Quantum Science and Technology Theory, Swinburne University of Technology, Melbourne, Victoria 3122, Australia*

@*School of Physics and Astronomy, Monash University, Melbourne, Victoria 3800, Australia*

△*ARC Centre of Excellence in Future Low-Energy Electronics Technologies, Monash University, Melbourne, Victoria 3800 Australia*

E-mail: pavel.kolesnichenko@alumni.uni-heidelberg.de; donatas.zigmantas@chemphys.lu.se

S1. Methods

Sample preparation

Monolayers of WS_2 were grown on sapphire (Al_2O_3) substrate via chemical vapor deposition (CVD) following a similar procedure described previously.[1] For TR-PEEM experiments, the monolayers were subsequently transferred onto an n-doped silicon wafer with a natural oxide layer (SiO_2/n-Si).

Electronic-band-structure calculation

Electronic band structure of WS_2 monolayer was calculated using density functional theory (DFT) as implemented in Quantum Espresso.[2]

TR-PEEM experiment

Broadband visible excitation (pump) pulses (∼10 fs duration, ∼2 eV central energy, ∼320 meV spectral bandwidth, 2.7–44 pJ energy per pulse) were generated in a lab-built non-collinear optical parametric amplifier (NOPA). Broadband deep-UV ionization (probe) pulses (∼10 fs duration, ∼4.7 eV central energy, ∼330 meV spectral bandwidth, 7 pJ energy per pulse) were generated as second harmonic of the output (with 2.34 eV central energy) from a second NOPA via achromatic phase matching.[3,4] Pulse repetition rate was 100 kHz. Both the excitation and ionization beams were weakly focused onto the sample inside the PEEM vacuum chamber at an angle of 25° with respect to the sample surface. Both beams were p-polarized and had ellipse-shaped spots on the sample surface with long and short axes being ∼130 μm and ∼80 μm (estimated fwhm) in the case of pump beam, and ∼200 μm and ∼50 μm in the case of probe beam, respectively. The pump and probe fluences at the sample were in the range of 8–140 nJ/cm^2 and 70 nJ/cm^2, respectively. The power in the probe beam was chosen low enough for samples not to degrade over the course of experiments[5] as well as for images to be acquired without space charge effects,[6] but sufficiently high to be able to

observe prominent pump-induced dynamics. Integration times during signal acquisition for each pump-probe delay were 40 *sec* (for 8 and 17 nJ/cm^2 pump), 20 *sec* (for 34 nJ/cm^2 pump), and 10 *sec* (for 140 nJ/cm^2 pump). Samples were contained in ultrahigh vacuum ($\sim 10^{-10}$ *mbar*) at 293 *K* inside a commercial PEEM apparatus (IS-PEEM, Focus GmbH) where electrons photoemitted from the sample ultimately formed an image on a charge-coupled device (CCD). The temporal and spatial resolution of the setup was estimated to be ∼75 *nm* and ∼13 *fs*, respectively (Figures S1,S2).

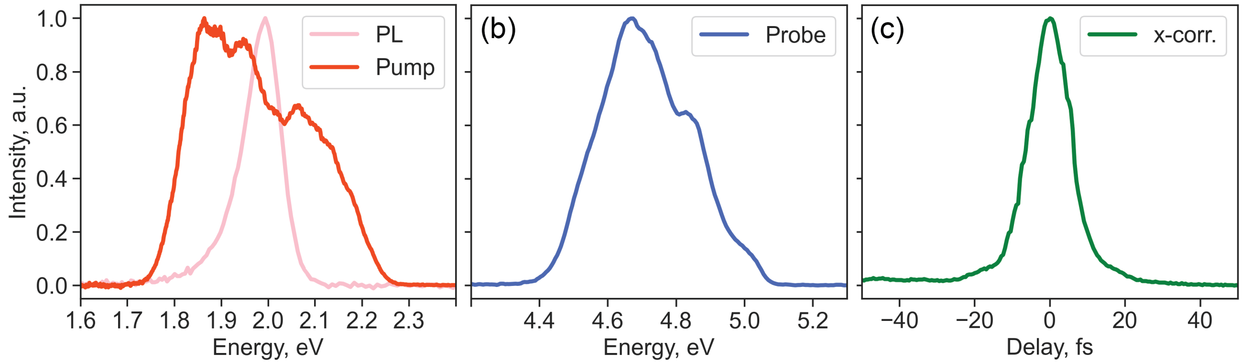

Figure S1: (a) Pump and (b) probe spectra, and (c) temporal cross-correlation (x-corr.) of pump and probe pulses measured via pump-induced transient-grating gate in a thin fused silica plate.[4] The x-corr. was measured in front of the PEEM's input window taking the window material into account. The fwhm of x-corr. profile is 13.4 *fs* defining temporal resolution of the TR-PEEM apparatus. In (a), a normalized photoluminescence (PL) spectrum of a WS_2 monolayer on n-doped Si is shown in pink.

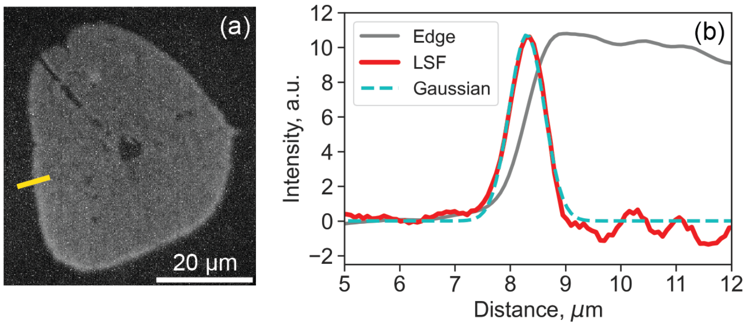

Figure S2: (a) Monolayer WS_2 flake imaged via a mercury lamp. (b) Edge profile (grey) taken along the yellow line indicated in (a), line spread function (LSF, red) of the edge profile, and a Gaussian fit to LSF with fwhm of 74.6 *nm* defining spatial resolution of the TR-PEEM apparatus.

S2. Pump-induced excitation and photoemission

Table S1: Pump fluences and corresponding carrier excitation densities.

Pump fluence, nJ/cm^2	8	17	34	140
Excitation density, $10^{11} \times cm^{-2}$	0.52	1.05	2.12	8.50

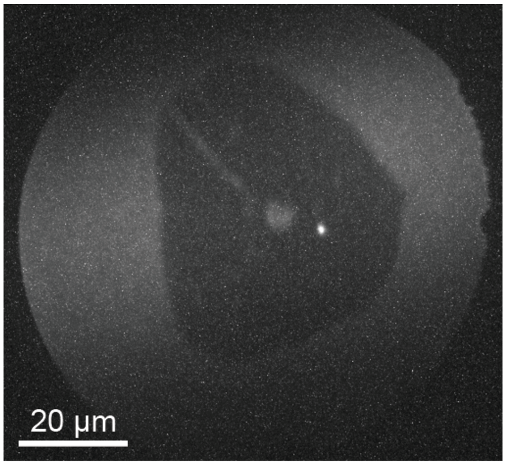

Figure S3: A photoemission image of the WS_2 monolayer flake obtained using 140 nJ/cm^{-2} pump pulses (with probe pulses blocked).

S3. Monolayer-specific dynamics

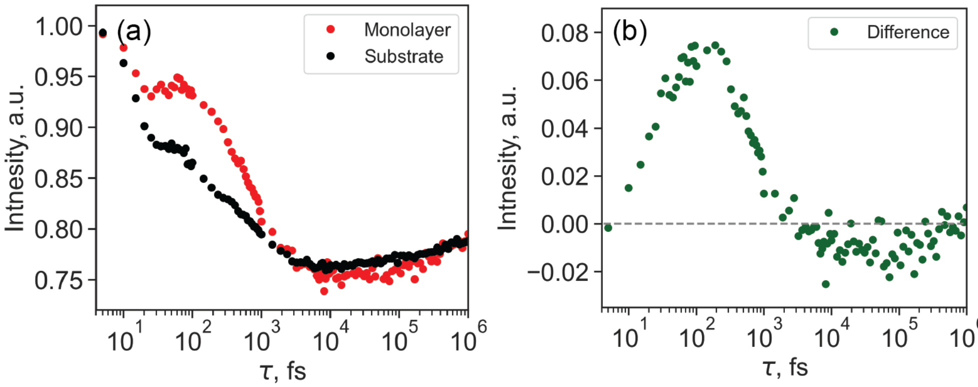

Figure S4: (a) Normalization of raw pump-probe traces obtained from WS_2 monolayer interior and substrate. (b) Difference (also Figure 2a of the main text) between the pump-probe traces in (a). Pump fluence was 34 nJ/cm^2.

The two spectra were pinned at 0 fs and 1 ns delays (Figure S4a) and subtracted (Figure S4b). This procedure naturally suppresses the effects from pulse overlap including coherent exciton population signal.

S4. Fittings of pump-probe traces

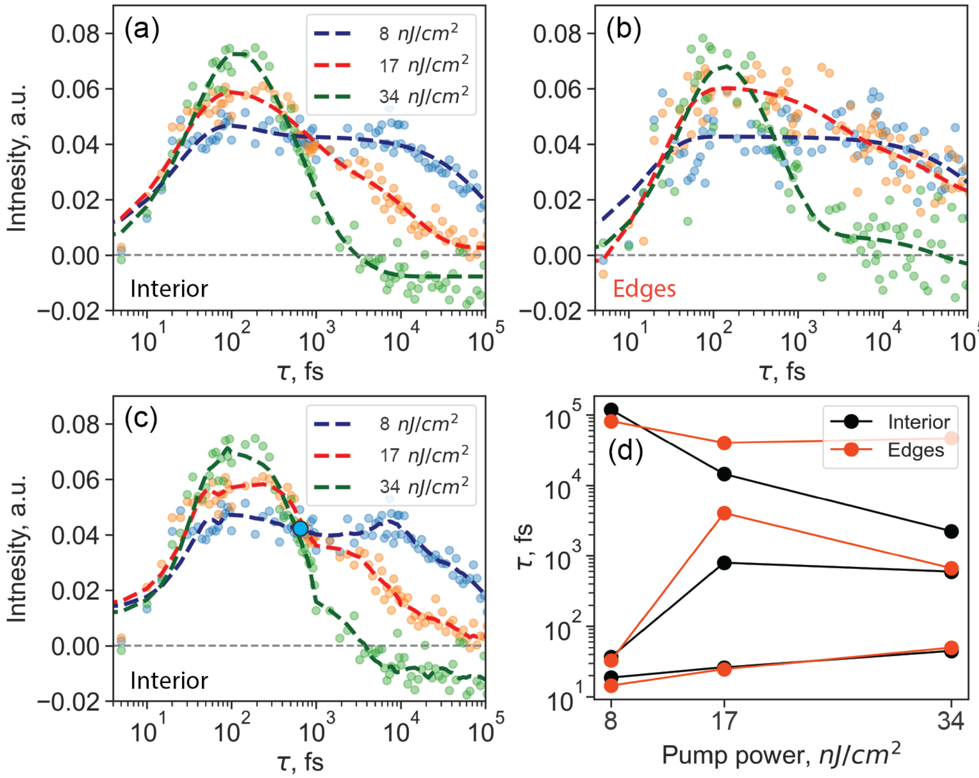

Figure S5: Fittings of the pump-probe traces acquired from the interior (a) and edges (b) of the monolayer flake for three pump fluences. (c) A result of smoothing of pump-probe traces revealing fine details such as a secondary rising signal. (d) Dependence of extracted time constants on pump fluence.

Table S2: Extracted time-constants for the three pump fluences (interior of the flake)

	$8\ nJ/cm^2$	$17\ nJ/cm^2$	$34\ nJ/cm^2$
$\tau_{rise},\ fs$	18.7	26.3	45.1
$\tau_1,\ fs$	36.9	797	596
$\tau_2,\ ps$	118.2	14.5	2.2

Table S3: Extracted time-constants for the three pump fluences (flake's edges)

	$8\ nJ/cm^2$	$17\ nJ/cm^2$	$34\ nJ/cm^2$
τ_{rise}, fs	14.5	24.6	50.0
τ_1, fs	32.7	2747	624
τ_2, ps	81.6	40.2	46.6

S5. Photoemission contrast drop for higher pump fluences

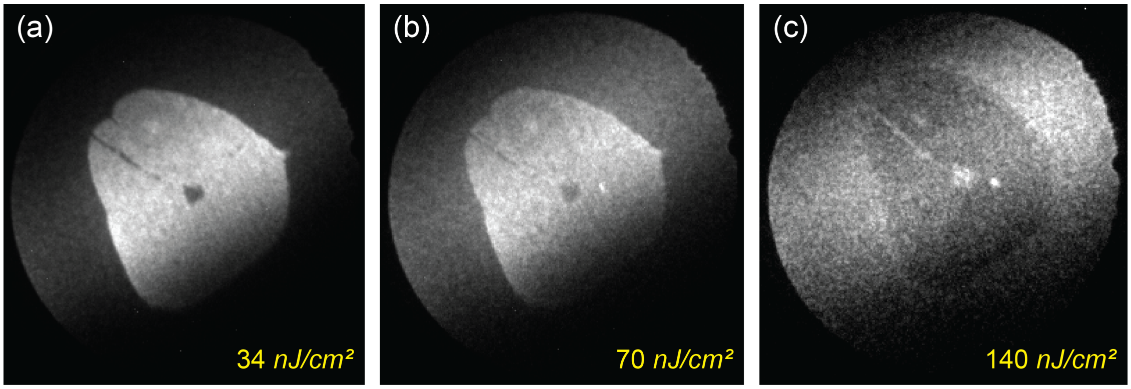

Figure S6: Photoemission at 7 ps pump-probe delay for three pump fluences of (a) 34 nJ/cm^2, (b) 70 nJ/cm^2, and (c) 140 nJ/cm^2. For higher fluences photoemission contrast from the flake becomes lower.

S6. Fitting with two rise-and-decay signals

Extracted rise time-constants are $\tau_{rise,1} \sim 23.7$ fs and $\tau_{rise,2} \sim 1.1$ ps comparable to previously reported values of dark-exciton[7,8] and trion formation.[9]

$$I = \left((1 - a_{rise,1} e^{-\frac{\tau - \tau_{0,1}}{\tau_{rise,1}}}) \cdot a_1 e^{-\frac{\tau - \tau_{0,1}}{\tau_1}} + (1 - a_{rise,2} e^{-\frac{\tau - \tau_{0,2}}{\tau_{rise,2}}}) \cdot a_2 e^{-\frac{\tau - \tau_{0,2}}{\tau_2}} + a_0 \right) \cdot H(\tau - \tau_0) \tag{S1}$$

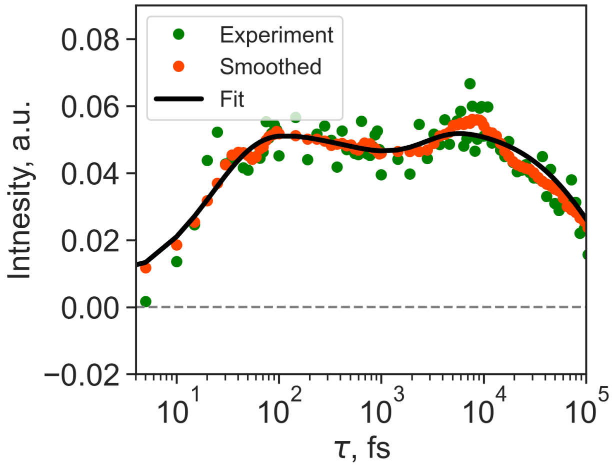

Figure S7: Fitting with a model (Eq. (S1)) accounting for two rise-and-decay signals. Pump fluence is 8 nJ/cm^2.

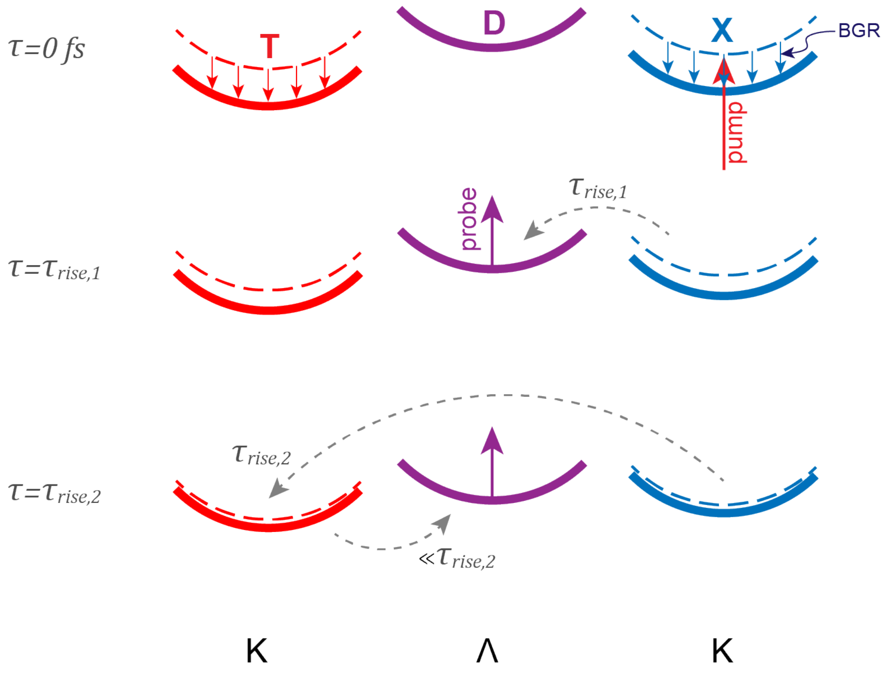

Figure S8: A possible trion-formation scheme. X = excitons; T = trions; D = dark carriers and/or defects; BGR = bandgap renormalization; τ = pump-probe delays; K, Λ are symmetry points in the first Brillouin zone. Here, for clarity, X and T valleys were separated and decay channels omitted.

A possible trion-formation scheme resulting in the increase of the photoemission signal (stimulated by probe pulses) is shown in Figure S8. During the pump-probe overlap a direct-exciton reservoir (X) is created and at the same time bandgap renormalization (BGR) occurs.

A fraction of these excitons scatter and decay leading to their depopulation, which translates to gradual recovery of the bandgap towards its unperturbed value. Excitons scattered to Λ-valleys become dark and yield a photoemission signal appearing with the delay of $\tau_{rise,1}$. Dark-exciton decay and/or back-scattering away from Λ-valleys occurring before $\tau_{rise,2}$ result in the reduction of the photoemission signal. About $\tau_{rise,2}$ later, trions form and at the same time their energy level becomes ever-closer to the minima of the Λ-valleys increasing K-Λ scattering probability. Such scattering occurring on timescales $\ll \tau_{rise,2}$ is rate-limited by $\tau_{rise,2}$ resulting in a secondary photoemission wave occurring with the same delay.

S7. Comment on other possible effects

One of the possible additional contributions to the measured signal could originate from the finite formation times of bright excitons. Very high time resolution of 13 fs in our experiments resulted in the broad spectrum that had significant spectral amplitude of the excitation at the energies up to 180 meV above the main exciton transition. This could potentially imprint bright-exciton formation times into the extracted τ_{rise} of dark excitons. We note, however, that exciton thermal relaxation and formation in 2D materials is extremely fast owing to enhanced electron-hole Coulomb interactions. Thus, Trovatello *et al.*[10] showed that, in the case of MoS_2 monolayers, exciting 430 meV above the main exciton resonance leads to the exciton formation times of < 10 *fs*. In our case, the spectrally broad pump excites the WS_2 monolayer up to ~180 meV above the main resonance, which is projected to yield bright-exciton cooling and formation time on even faster time scale. Therefore, we do not expect any delayed signal originating from bright exciton formation to be resolved and/or convoluted with intervalley scattering.

Another contribution to the measured dynamics could originate from the intervalley scattering of spin-forbidden excitons. Indeed, it has been shown that in monolayers of WS_2, the spin-flip dynamics occurs on a ~100 fs timescale at room temperature.[7] These

dynamics, although fast, are several times slower than intervalley scattering, meaning that a lesser fraction of bright excitons will be intravalley-scattered into spin-forbidden dark excitons and a larger fraction of bright excitons will be intervalley-scattered into momentum-forbidden dark excitons. This lesser number of spin-forbidden dark excitons could potentially contribute to the photoemission response via secondary, intervalley, scattering to Λ-valleys. Since their energy is lower than that of bright excitons, this scattering should also be slower than direct intervalley scattering of bright excitons. We have not detected any time constants that would be $\sim$100 fs in our experiments. In addition, due to the second-order character of spin-dark-exciton intervalley scattering process, its contribution should be much smaller than the first-order scattering of bright excitons. Furthermore, the energetic barrier between the spin-dark states at K symmetry points and the corresponding adjacent same-spin states at Λ points is sufficiently large thus forbidding natural spin-flow between these states.[11] This further diminishes the likelihood of the detection of spin-forbidden dark excitons in TR-PEEM experiments. Therefore, overall, we believe that although spin-forbidden dark excitons can find their way to the Λ-valleys their contribution to pump-probe photoemission response should be negligible.

Finally, due to the likely residual doping in CVD-grown WS_2 monolayers, the doping electrons could also contribute their dynamics into the measured pump-probe signal. These conduction band electrons, which are distributed across all valleys that are below the Fermi energy, can indeed be excited and photoemitted. However, since the photoemission horizon minimum is below the conduction band minimum and the conduction electrons are initially in thermal equilibrium, we do not expect any discernible fast dynamics to be detected from these electrons but rather, perhaps, a slowly varying pump-probe temporal background.

References

1. Zhang, Q.; Lu, J.; Wang, Z.; Dai, Z.; Zhang, Y.; Huang, F.; Bao, Q.; Duan, W.; Fuhrer, M. S.; Zheng, C. Reliable Synthesis of Large-Area Monolayer WS_2 Single Crystals, Films, and Heterostructures with Extraordinary Photoluminescence Induced by Water Intercalation. *Advanced Optical Materials* **2018**, *6*, 1701347.

2. Giannozzi, P.; Baroni, S.; Bonini, N.; Calandra, M.; Car, R.; Cavazzoni, C.; Ceresoli, D.; Chiarotti, G. L.; Cococcioni, M.; Dabo, I.; Dal Corso, A.; de Gironcoli, S.; Fabris, S.; Fratesi, G.; Gebauer, R.; Gerstmann, U.; Gougoussis, C.; Kokalj, A.; Lazzeri, M.; Martin-Samos, L. *et al.* QUANTUM ESPRESSO: a modular and open-source software project for quantum simulations of materials. *Journal of Physics: Condensed Matter* **2009**, *21*, 395502.

3. Baum, P.; Lochbrunner, S.; Riedle, E. Tunable sub-10-fs ultraviolet pulses generated by achromatic frequency doubling. *Optics Letters* **2004**, *29*, 1686.

4. Bruder, L.; Wittenbecher, L.; Kolesnichenko, P. V.; Zigmantas, D. Generation and compression of 10-fs deep ultraviolet pulses at high repetition rate using standard optics. *Optics Express* **2021**, *29*, 25593.

5. Li, Y.; Liu, W.; Wang, Y.; Xue, Z.; Leng, Y.-C.; Hu, A.; Yang, H.; Tan, P.-H.; Liu, Y.; Misawa, H.; Sun, Q.; Gao, Y.; Hu, X.; Gong, Q. Ultrafast Electron Cooling and Decay in Monolayer WS_2 Revealed by Time- and Energy-Resolved Photoemission Electron Microscopy. *Nano Letters* **2020**, *20*, 3747–3753.

6. Buckanie, N. M.; Göhre, J.; Zhou, P.; von der Linde, D.; Horn-von Hoegen, M.; Meyer zu Heringdorf, F.-J. Space charge effects in photoemission electron microscopy using amplified femtosecond laser pulses. *Journal of Physics: Condensed Matter* **2009**, *21*, 314003.

7. Wang, L.; Xu, C.; Li, M.-Y.; Li, L.-J.; Loh, Z.-H. Unraveling Spatially Heterogeneous

Ultrafast Carrier Dynamics of Single-Layer WSe_2 by Femtosecond Time-Resolved Photoemission Electron Microscopy. *Nano Letters* **2018**, *18*, 5172–5178.

8. Wallauer, R.; Perea-Causin, R.; Münster, L.; Zajusch, S.; Brem, S.; Güdde, J.; Tanimura, K.; Lin, K.-Q.; Huber, R.; Malic, E.; Höfer, U. Momentum-Resolved Observation of Exciton Formation Dynamics in Monolayer WS_2. *Nano Letters* **2021**, *21*, 5867–5873.

9. Singh, A.; Moody, G.; Tran, K.; Scott, M. E.; Overbeck, V.; Berghäuser, G.; Schaibley, J.; Seifert, E. J.; Pleskot, D.; Gabor, N. M.; Yan, J.; Mandrus, D. G.; Richter, M.; Malic, E.; Xu, X.; Li, X. Trion formation dynamics in monolayer transition metal dichalcogenides. *Physical Review B* **2016**, *93*, 041401(R).

10. Trovatello, C.; Katsch, F.; Borys, N. J.; Selig, M.; Yao, K.; Borrego-Varillas, R.; Scotognella, F.; Kriegel, I.; Yan, A.; Zettl, A.; Schuck, P. J.; Knorr, A.; Cerullo, G.; Conte, S. D. The ultrafast onset of exciton formation in 2D semiconductors. *Nature Communications* **2020**, *11*, 5277.

11. Godiksen, R. H.; Wang, S.; Raziman, T. V.; Rivas, J. G.; Curto, A. G. Impact of indirect transitions on valley polarization in WS_2 and WSe_2. *Nanoscale* **2022**, *14*, 17761–17769.
